# Detection of Circulating Tumor Cells

**DOI:** 10.1155/2014/819362

**Published:** 2014-07-15

**Authors:** Sanne de Wit, Guus van Dalum, Leon W. M. M. Terstappen

**Affiliations:** Department of Medical Cell BioPhysics, Faculty of Science and Technology, MIRA Institute for Biomedical Technology and Technical Medicine, University of Twente, Carre, Room C4437, Hallenweg 23, 7522 NH Enschede, The Netherlands

## Abstract

The increasing number of treatment options for patients with metastatic carcinomas has created an accompanying need for methods to determine if the tumor will be responsive to the intended therapy and to monitor its effectiveness. Ideally, these methods would be noninvasive and provide quantitative real-time analysis of tumor activity in a variety of carcinomas. Assessment of circulating tumor cells shed into the blood during metastasis may satisfy this need. Here we review the CellSearch technology used for the detection of circulating tumor cells and discuss potential future directions for improvements.

## 1. Introduction

In 1869, Thomas Ashworth described the microscopic observation of circulating tumor cells (CTC) in the blood of a man with metastatic cancer. He concluded that the CTC must have passed through the circulatory system to arrive at the vein from which the blood was collected [1]. The critical role that circulating tumor cells play in the metastatic spread of carcinomas has been demonstrated more than 100 years later [2]. Only recently technology has become available with the requisite sensitivity and reproducibility to explore the diagnostic potential of CTC [3].

Via a rigorous clinical testing program, CellSearch is the only system validated for CTC detection to date [4–10]. The device is cleared by the FDA for the monitoring of patients with metastatic breast, colorectal, and prostate cancer and clinical utility has also been demonstrated in metastatic small and non-small cell lung cancer [11, 12], stomach cancer [13], pancreas cancer [14], ovarian cancer [15], and bladder cancer [16–18].

For the enumeration of CTC, the CellSearch reagent kit uses ferrofluids labeled with the epithelial cell adhesion molecule (EpCAM), a DNA dye to stain nuclei and antibodies to target CD45 and cytokeratin 8, 18, and 19. The enrichment of endothelial and melanoma cells was enabled by replacing EpCAM ferrofluids with CD146 ferrofluids in the CellSearch system. Replacement of cytokeratin antibodies with CD105 allowed the enumeration of endothelial cells and studies showed an increase in endothelial cells in metastatic cancer and cardiovascular diseases [19–21]. Replacement of cytokeratin antibodies with antibodies to high molecular weight melanoma antigen allowed the enumeration of melanoma cells and their presence is associated with a poor prognosis [22].

The potential to assess the presence of treatment targets in CTC such as Bcl-2 [23], Her-2 [24, 25], AR [26], and IGFR1 [27] at both the DNA and protein level by the CellSearch system has spurred the interest in this field as it holds the promise of a “real time liquid biopsy.”

## 2. Cancer and the Formation of Metastasis

In the USA, 1.7 million people are expected to be diagnosed with cancer and 0.6 million people are expected to die from cancer [28]. At present, cancer is the second leading cause of mortality in USA and Europe [28, 29]. Although the 5-year relative survival rate for all cancers is improving (49% in 1975–1977 and 68% in 2002–2008), the number of people diagnosed with cancer is expected to increase due to the increase in age of the overall population. The improvement in survival reflects both progress in diagnosing certain cancers at an earlier stage and improvements in treatment. The costs associated with these improvements are however also increasing and will have an enormous economic impact in the time to come.

**Figure 1:**
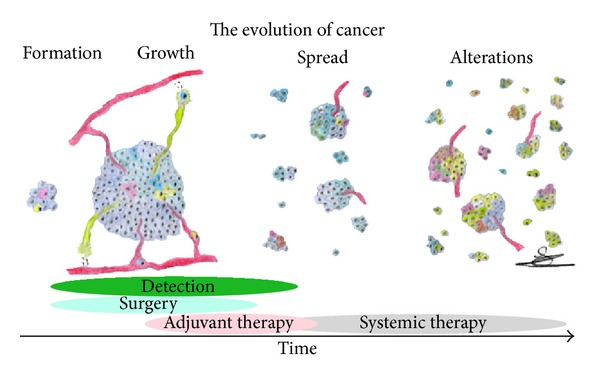
The evolution of cancer. After initial formation of cancer cells, growth of the tumor attracts blood vessels to supply oxygen and nutrients. Cancer cells then spread via these vessels forming metastases at distant sites. Mutations in DNA result in a heterogeneous population of cancer cells, with the potential of an increase in resistance against medicine. Patient care is depicted during the time of this evolution.

Death of cancer patients is rarely caused by the primary tumor and can be contributed in most cases to metastases at distant sites. Understanding the metastatic process is therefore of utmost importance to get more insight into the prognosis of patients and to identify potential ways to prevent tumors to form metastases.  Figure 1 illustrates the evolution of cancer. At the early stages of tumor cell formation, diversity of the tumor cells already occurs and some will gain a greater ability than other cells to expand (tumor stem cells). At the time a tumor reaches ~100 *µ*m in diameter, its need for nutrients increases. This is supplied through neovascularization, which permits the tumor to grow. At this time, cells from the tumor can enter the blood either directly or through the lymphatic system. Although the majority of these cells will succumb, some will survive and either passively or actively penetrate the endothelial cell layer at different sites in the body, forming distant metastasis that ultimately will kill the patient.

Cancers have preferences for certain tissues to form metastasis. The mechanisms and antigens expressed on their cell surface and the ligands on the capillaries of that specific tissue are still poorly understood. As time passes, the diversity of tumor cells increases, making the treatment more difficult. Moreover, the diversity further increases under the influence of therapy as tumor cells become resistant to therapy. Today, the potential sensitivity of a tumor is assessed on tumor cells taken at the time of surgery. In cases that the tumor has not been completely irradiated from the body tumor cells, tumor cells will remain dormant or will expand. At the time the tumor cells have formed a detectable metastasis, the cells may no longer have the same sensitivity to therapies as at the time of surgery. This makes it again necessary to obtain a tumor biopsy and assess the best treatment options. However, biopsies are difficult, if not impossible, to take from metastatic sites. The ability to isolate tumor cells from the blood provides a unique opportunity for a “real time liquid biopsy.” Of course, detection of cancer before dissemination has taken place is preferred. However, to make this possible, a leap in technology development is required. It has been modeled that tumors are very small at the moment of dissemination, and traditional imaging techniques need to be improved to detect these small tumors [30]. Also, to detect CTC in such early disease conditions, sensitivity of these tests will need to be improved significantly [30].

## 3. Identification of CTC by the CellSearch System

The CellSearch system (Janssen Diagnostics, LLC; Raritan, NJ) consists of the CellTracks Autoprep, CellTracks Magnest, CellSearch Epithelial Cell Kit, and the CellTracks Analyzer II. The reagent kit used for the enumeration of CTC (CellSearch Epithelial Cell Kit) contains ferrofluids labeled with EpCAM to select for cells of epithelial origin, the staining reagents 4′,2-diamidino-2-phenylindole, dihydrochloride (DAPI) for a nuclear stain, CD45-allophycocyan (CD45-APC) to label leukocytes, cytokeratin 8, 18 Phycoerythrin, and cytokeratin 19 Phycoerythrin (CK-PE) to label cells of epithelial origin, and buffers to enhance cell capture and permeabilize and fix the cells [31, 32]. Samples that will be processed up to 96 hours after collection are drawn into 10 mL evacuated blood draw tubes (Janssen Diagnostics, LLC; Raritan, NJ) and maintained at room temperature.

To obtain viable CTC or investigate the expression of RNA in CTC, blood should be collected in EDTA and preferably processed within 24 hours. For these experiments the CellSearch Profile Kit (Janssen Diagnostics, LLC; Raritan, NJ) should be used. In this kit epithelial derived cells are enriched by the use of ferrofluids labeled with antibodies targeting the EpCAM antigen. After processing with the CellTracks Autoprep, a cell suspension is obtained including the CTC and ~5000 residual leukocytes. This number will increase with the age of the blood samples. These samples can be used to investigate the mRNA expression of CTC or analyzed at the single cell level after staining and sorting by, for example, flow cytometry [33, 34].

**Figure 2:**
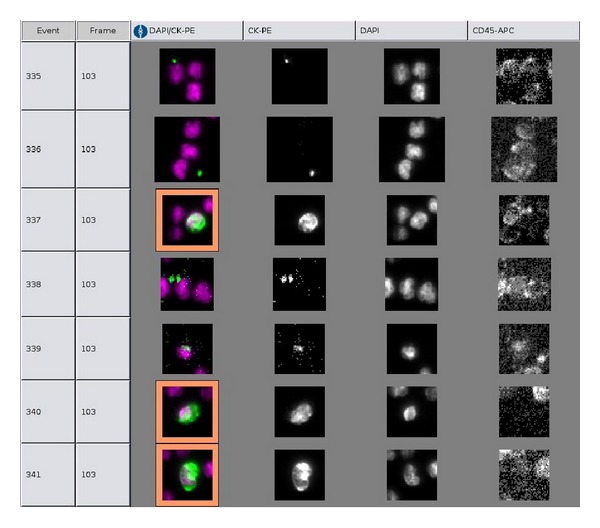
CellSearch thumbnail gallery. The software of the CellSearch CellTracks displays thumbnails of all objects that are positive for both DAPI and CK. Events 337, 340, and 341 show a CTC: positive for DAPI and PE and negative for CD45. Note the weak CD45-staining of several white blood cells in events 340 and 341.

The CellTracks Autoprep immunomagnetically enriches cells expressing EpCAM from 7.5 mL of blood and fluorescently labels the enriched cells with DAPI, CD45-APC, and CK-PE. The resuspended cells are deposited in the cartridge that is positioned in the CellTracks Magnest. This semiautomated fluorescence-based microscopy system acquires images using a 10X NA0.45 objective with filters for DAPI, PE, APC, and FITC (not used) to cover the complete surface area of the analysis chamber. A computer identifies objects staining with DAPI and PE in the same location and generates images for the DAPI, PE, APC, and FITC filters.  Figure 2 shows a typical display of the fluorescent images that passed the threshold set by the computer program. A reviewer selects the CTC defined as nucleated DAPI+ cells, lacking CD45 and expressing CK-PE from the gallery of objects, which are tabulated by the computer. After processing 7.5 mL of blood from healthy donors, the median number of objects that need to be scored is ~50. In blood samples from cancer patients, the number of objects can be quite large. In general these are not all CTC, but can mostly be contributed to the presence of CTC fragments [35, 36]. Presence of these CTC fragments is also related to poor outcome [36]. The heterogeneity in morphology is partly caused by the large diversity in the viability or apoptotic stage of the CTC. This makes it difficult to set criteria of what accounts as a CTC. Differences in assigning objects as CTC are the largest error currently in the system, and extensive training is required to keep the variations in assigning objects as CTC to a minimum [37, 38]. Recently, we developed a CTC detection algorithm that counts CTC in images recorded by the CellSearch system [39]. This algorithm used survival data of metastatic prostate cancer patients to arrive at a definition that optimally stratified the patients into groups with favorable and unfavorable survival. It was not developed to copy human reviewers that assign events, but it eliminates reviewer variability. In addition, it is fast and decreases the cost of the CTC assay by eliminating the time a reviewer spends on reviewing the images. Also, quantitative information can be derived about the objects counted as CTC, such as morphological features or quantitative expression of antigens expressed on the CTC [24, 40].

## 4. Frequency of CTC Detected by the CellSearch System

The number of cells with features that are consistent with those of CTC detected with the CellSearch system in 7.5 mL of blood from healthy donors or patients with nonmalignant diseases is remarkably low [3]. Lowering the stringency of the criteria to assign cells or objects increases the number of CTC detected in both controls and patients [36, 39]. The limited number of controls tested and less strict criteria to assign objects as CTC are an important reason for the high number of CTC reported by new technologies for detection of CTC. In fact, our earlier work used flow cytometry as the platform to analyze the immunomagnetically enriched samples and the number of CTC detected in both controls and patients was clearly higher. This can be contributed to the less stringent criteria, such as a no-cell morphology criterion [41, 42].

**Figure 3:**
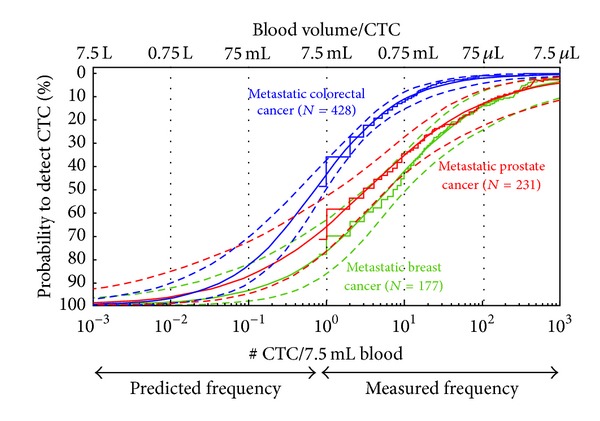
Frequency of CTC in metastatic colorectal prostate and breast cancer. Frequency was measured in 7.5 mL of blood (right half of the figure) and predicted in larger blood volumes (left half of the figure). Extrapolation of number of CTC was performed by a log-logistic function (solid line) including 95% confidence interval (dashed lines) and fitted through the empirical cumulative distribution functions (stair plots) for metastatic breast, colon, and prostate cancer. The fitted curve shows the blood volume that is needed (7.5 L) to detect the presence of CTC in all patients (100% probability) in a metastatic setting, using the CellSearch approach. Adapted figure from [46].

Many new studies have reported the frequency of CTC detected by the CellSearch system, since the original report on the frequency of CTC detected with the CellSearch system in controls and patients with a variety of carcinomas [3].  Table 1 provides a summary of the frequency of CTC at various thresholds reported in these studies in several carcinomas, healthy donors, and patients with nonmalignant diseases. If CTC are to be used for the assessment of treatment targets to choose the most appropriate therapy, sufficient number of CTC will need to be available for detailed analysis. The heterogeneity of the tumor cells forces one to examine multiple individual cells and a minimum of 10–100 cells seems reasonable [25, 26, 43–45].  Table 1, however, shows that the number of patients (*n*) with sufficient number of CTC in 7.5 mL of blood for this purpose is very low. Therefore, the number of CTC in larger volumes of blood was estimated by fitting the frequency distribution of CTC present in 7.5 mL of blood [46].  Figure 3 shows the frequency distribution of CTC detected in 7.5 mL of blood by the CellSearch system in patients with metastatic breast cancer (stair plot green line), metastatic colorectal cancer (stair plot blue line), and metastatic prostate cancer (stair plot red line). The solid lines show the best fit for this distribution and the dotted line is the 95% confidence level around this distribution. This figure shows that a 100-fold increase in blood volume is needed to detect CTC in all patients. All the blood will need to be analyzed to obtain sufficient number of CTC for characterization and guidance of therapy.

## 5. Relation between Presence of CTC and Survival

**Figure 4:**
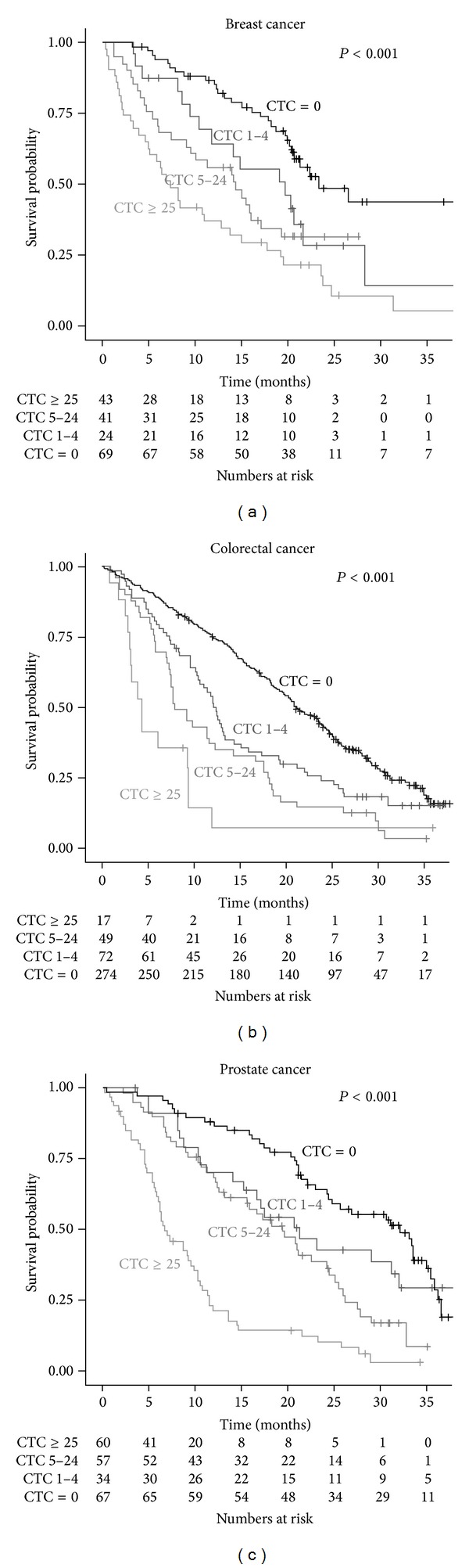
Kaplan-Meier plots of samples from metastatic breast (a), colon (b), and prostate (c) cancer patients with 0, 1–4, 5–24, and >25 CTC at the start of therapy. The number of patients at risk is listed at every time point of measurement.

The presence of CTC is associated with a relative poor prognosis. This was demonstrated in prospective multicenter studies in metastatic colorectal cancer [8], prostate cancer [10], and breast cancer [4]. A discrimination between patients with favorable CTC (<3 for colorectal cancer or <5 for breast and prostate cancer) and unfavorable CTC (≥3 or ≥5) was made in the original papers reporting the results of these studies. In practice, a further discrimination in patients with unfavorable CTC can be made when the actual peripheral blood tumor load is considered. This is illustrated by the Kaplan Meier plots in Figure 4. Blood is drawn before starting a new line of therapy and patients are divided into categories with 0 CTC, 1–4 CTC, 5–24 CTC, and >25 CTC. The difference in survival curves becomes larger after the first cycles of therapy, as the CTC in those patients benefitting from therapy are eliminated. A guide for the interpretation of changes in CTC is described in detail elsewhere [67]. Altogether, it is clear that all CTC will need to be eliminated for a treatment to be truly effective and prolong survival of the patient.

## 6. Challenges in CTC Identification

The potential of CTC detection and characterization has stimulated the interest of many investigators to develop new CTC platforms [68–81]. The challenge in identifying CTC lies in the detection of these rare cells in blood. In metastatic cancer patients, approximately 1 CTC per mL blood will be surrounded by approximately 5 · 10^6^ white blood cells and 5 · 10^9^ red blood cells [3, 46]. Differences in the approaches taken to enrich and detect CTC have been reviewed extensively elsewhere [82–85].

**Figure 5:**
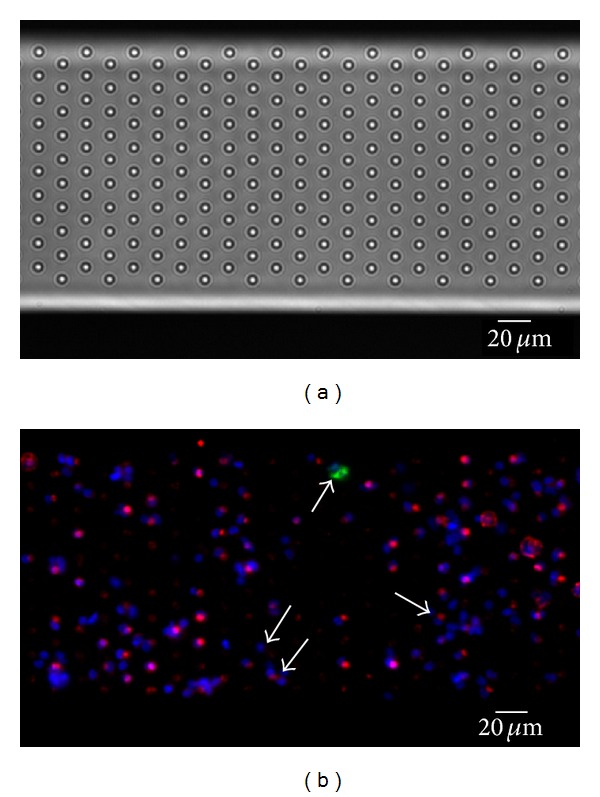
Cells from CellSearch Waste immunostained on a microsieve. Blood from a lung cancer patient was used for a CellSearch assay. After immunomagnetic selection, part of the sample was discarded by the system and used for filtration on a microsieve with 5 *µ*m pores. Bright-field image of the sieve is shown in (a). (b) shows the sieve with filtered sample. Cells were stained for nucleus (blue), cytokeratin C11 (green), and CD45 (red). Fat arrow points to a CTC, positive in CK. Small arrows point to the absent staining of cells, showing the difficulty of accounting for all cells on the sieve. Image taken on a fluorescence microscope with a 10x (0.45NA) objective.

One of the approaches we are currently evaluating is filtration of blood to detect CTC that have a relatively large size and stiffness compared to blood cells [86, 87]. In the optimization of this approach, we envisioned the ideal filter for CTC enrichment to be constructed of a stiff, flat material that is impervious to blood cells. To effectively pass blood collected in CellSave tubes, at least 100,000 regularly spaced 5 *µ*m pores with a low porosity are needed [71, 88]. To determine whether CTC have escaped the EpCAM immunomagnetic detection in CellSearch, we constructed a device that collects the blood discarded by the system after immunomagnetic selection of EpCAM+ cells [87, 89]. This blood, lacking EpCAM+ cells, is then passed through a 36 mm^2^ microsieve with 111,800 5 *µ*m pores. The cells on the filter are immunostained to distinguish CTC from non-CTC and examined by fluorescent microscopy.  Figure 5 shows an example of a microsieve; the upper panel shows a bright-field image of a section of a microsieve and the lower panel shows an overlay of fluorescent images of the nucleic acid dye DRAQ5 (blue), CD45-Brilliant Violet staining (red), and cytokeratin-PE staining (green). In the image, a CTC of a lung cancer patient is visible among many other cells. The figure also shows that not all nuclei stain with CD45 or cytokeratin. Currently, efforts are ongoing to identify the tissue of origin of these nonidentified cells on the microsieve. Either these cells could still be leukocytes that lost the CD45 antigen or the fluorophore Brilliant Violet does not emit sufficient light to be detected, or the cell is damaged and lost its cytoplasmic membrane. Other alternative explanations may be that these cells are not of hematopoietic lineage, such as endothelial cells, or that these are CTC that do not express the cytokeratins that are recognized by the C11 clone used to stain the cytokeratins. This lack of cytokeratin expression could be a result of the epithelial-mesenchymal transition (EMT) process [90].

Besides cytokeratins, EpCAM expression is used in the majority of CTC enrichment methods based on antibody-capture [91, 92]. Yet EMT could downregulate this protein and other epithelial proteins, leading to a subpopulation of CTC that will be missed during enrichment or detection. CTC that are partially in EMT can coexpress mesenchymal proteins, like vimentin, N-cadherin, and O-cadherin [93, 94]. The CellSearch system only uses a limited panel of cytokeratins for detection and changes in cytokeratin expression during EMT can therefore influence the CTC detection. An expanded panel of cytokeratins is of interest for complete detection and is applied in our search for EpCAM− cells after filtration of the CellSearch waste. To find EpCAM− CTC subpopulations, novel antibodies are of increasing interest to be analyzed as an additional feasible selection marker. CTC populations with expression or lack of expression of epithelial and mesenchymal proteins characterize the complexity and heterogeneity of CTC. The major challenge in addressing these problems is that it is unknown whether CTC are present in the blood sample. If they are present, their heterogeneity of unknown extent is encountered. It requests an increasing diversity in CTC detection and characterization in current and future methods.

## 7. Assessment of Treatment Targets in CTC

As described earlier, identification of CTC in the CellSearch system uses EpCAM expression for immunomagnetic selection and subsequently DNA, CK, and CD45 staining for identification of the enriched cells. Less strict qualifications for CTC definitions, omitting, for instance, the DNA+ or CD45− qualification, increase the frequency of objects counted as CTC in patients and controls [46]. EpCAM+ CK+ CTC can be differentiated into intact CTC, CTC fragments, and CTC microparticles. The presence of all these is associated with a relatively short survival in castrate resistant prostate cancer [36]. However, intact CTC containing DNA can provide more information, as they are receptive to molecular and phenotypic characterization. RNA or DNA from CTC can offer a representation of the genetic composition of the tumor and may be especially useful when a tumor biopsy is unavailable. Cell sorting of CTC after CellSearch analysis showed that almost 45% of the exomes in single CTC could be sequenced and whole genome amplification allows for variant calling in single CTC [34].

**Figure 6:**
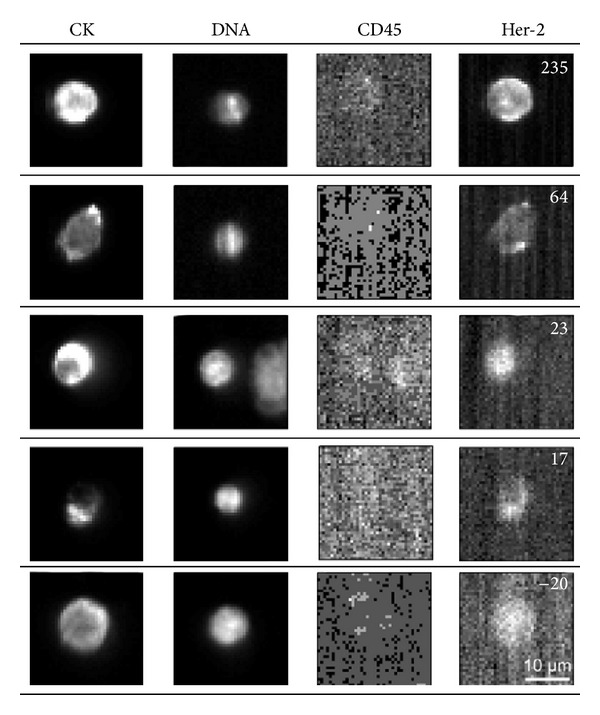
Example of five CTC from five different patients. Fluorescence of CTC Her-2 expression (right column) is quantified by the number in upper right corner. A higher positive number represents a higher Her-2 expression, whereas a negative number (bottom picture) represents no Her-2 expression on that CTC. The scale bar is applied to all images. Adapted figure from [24].

For breast cancer patients, status of the membrane protein Her-2 may guide their therapy and is of great value for personalized treatment. Usually, tumor biopsies taken at the time of surgery are analysed for their Her-2 status, but may not be representative for the tumor at the time of metastasis. CTC may circumvent this problem and allow real-time determination of the Her-2 status of the tumor. It can be subjective to determine whether or not a protein like Her-2 is expressed and at what level. Tools will be needed to quantify the actual expression levels to reliably investigate the relation to the response of therapy targeting the Her-2 receptor.  Figure 6 shows an example of an approach to quantify Her-2 expression on CTC. An automated algorithm is used to identify CTC and provides a numerical value to the level of Her-2 expression on CTC. It is quite obvious that the accuracy of Her-2 expression and the ability to assess its heterogeneity will improve with the number of CTC that are detected. Feasibility for assessment of treatment targets on CTC has been demonstrated for a variety of treatment targets at the protein and genetic level. This supports the notion that CTC indeed can be used to guide personalized therapy in the future, provided that CTC indeed can be isolated from the patient [23, 25–27, 34, 39, 45].

## 8. Outlook

**Figure 7:**
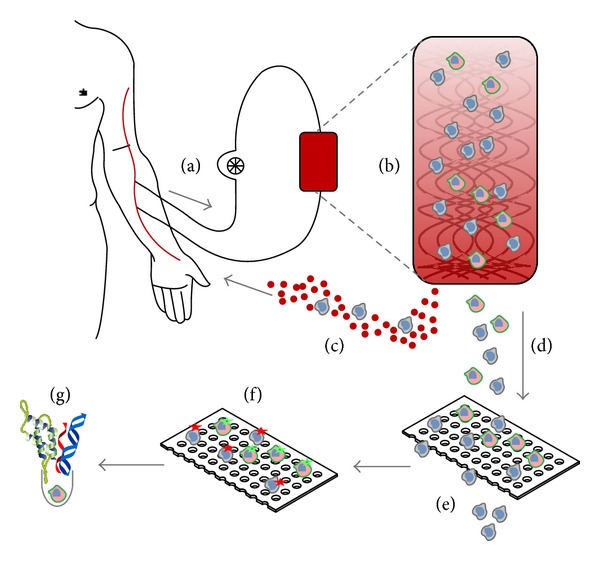
Schematic representation of the CTCTrap. Blood from a patient (a) is passed through a functionalized 3D matrix (b). The porous matrix can withstand up to 5 L of blood flow. In this matrix are one or more specific antibodies present for CTC capture. A continuous blood flow without cells of interest is circled back to the patient (c). Retained cells are eluted from the matrix (d) and will be filtered through 1–5 *µ*m pores to reduce hematopoietic background (e). Cells retained on the filter can be used for immunofluorescent staining to discriminate CTC from non-CTC (f) and subsequently be used for isolation of single CTC for additional molecular characterization, like protein, RNA, and DNA analysis (g).

Treatment of cancer is evolving from chemotherapy towards a more personalized approach, with drugs that recognize specific targets. To define the presence of specific targets, an analysis of the tumor is required at the start of therapy. CTC are likely representatives of the tumor to be treated and can therefore be used as a liquid biopsy. However, sufficient numbers of CTC are required to obtain a representative picture. To arrive at a sufficient number of CTC, a new approach is being explored by the European Consortium “CTC Therapeutic Apheresis” (http://www.utwente.nl/tnw/ctctrap/). The concept of this approach is presented in Figure 7. The CTCTrap combines immunocapture and size-based separation of CTC from their hematopoietic background. A large volume of blood is transported through a matrix and then reintroduced in the body, while CTC are captured in the matrix. After elution, CTC can be individually isolated for further characterization. This can, for example, assess the likelihood that certain therapies will be effective. The CTCTrap is expected to deliver a complete platform to capture, enumerate, and characterize CTC. Detection of all CTC in blood will change the current methods of diagnosis and treatment for patients with known and unknown metastatic disease.

## Figures and Tables

**Table 1:** Summary of CTC counts in 7.5 mL of blood from patients (*n*) with various types of metastatic carcinomas. It represents the percentage of patients (%) from the total group of patients (*n*) above a certain CTC cut-off, detected with the CellSearch System.

	% (*n*) ≥ 1		% (*n*) ≥ 2		% (*n*) ≥ 3		% (*n*) ≥ 5		% (*n*) ≥ 10		% (*n*) ≥ 50		% (*n*) ≥ 100		References
Subject															
Healthy	2	(330)	0.3	(330)	0	(185)	0	(330)	0	(330)	0	(330)	0	(330)	[4, 13, 47–49]
Nonmalignant	5	(398)	1	(398)	0	(101)	0	(101)	0	(101)	0	(101)	0	(101)	[4, 47]

Metastatic cancer type															
Bladder	47	(53)	35	(20)	—		25	(53)	5	(20)	0	(20)	0	(53)	[50, 51]
Breast	55	(200)	53	(562)	33	(91)	38	(671)	32	(562)	18	(268)	12	(562)	[4, 47, 52–55]
Colorectal	48	(545)	34	(455)	32	(676)	18	(455)	12	(455)	0	(42)	0	(455)	[8, 56–59]
Gastric	67	(27)	56	(27)	41	(27)	26	(27)	19	(27)	4	(27)	4	(27)	[13]
Lung, non-small cell	46	(57)	28	(117)	20	(20)	11	(57)	10	(20)	5	(20)	5	(20)	[53, 60, 61]
Lung, small cell	95	(38)	89	(62)	79	(38)	79	(38)	74	(38)	53	(38)	47	(38)	[12, 62]
Ovarian	—		14	(216)	—		—		—		—		—		[15]
Pancreatic	35	(72)	19	(72)	15	(72)	8	(72)	7	(72)	3	(72)	3	(72)	[63, 64]
Prostate	60	(149)	80	(40)	66	(95)	59	(314)	53	(40)	33	(40)	18	(40)	[10, 48, 49, 53, 65, 66]
